# Covered Self‐Expanding Metal Stents Versus Multiple Plastic Stents in Treating Biliary Strictures Post‐Orthotopic Liver Transplantation: A Systematic Review and Meta‐Analysis of Randomized Controlled Trials

**DOI:** 10.1002/deo2.70143

**Published:** 2025-05-23

**Authors:** Ygor Rocha Fernandes, Thiago Arantes de Carvalho Visconti, Marcelo Klotz Dall'Agnol, André Orsini Ardengh, Matheus de Oliveira Veras, Evellin Souza dos Santos Valentim, Marcos Eduardo Lera dos Santos, Sergio Eiji Matuguma, Wanderley Marques Bernardo, Eduardo Guimarães Hourneaux de Moura

**Affiliations:** ^1^ Department of Gastroenterology Hospital das Clínicas University of São Paulo Medical School (HCFMUSP) Sao Paulo Brazil; ^2^ Faculty of Medicine University of São Paulo Sao Paulo Brazil; ^3^ Medical Guidelines Program, Brazilian Medical Association Sao Paulo Brazil; ^4^ Gastrointestinal Endoscopy Unit Clinical Hospital of Faculty of Medicine of the University of São Paulo Sao Paulo Brazil; ^5^ Graduate Program in Gastroenterology Faculty of Medicine of the University of São Paulo Sao Paulo Brazil

**Keywords:** biliary stricture, covered self‐expanding metal stents, endoscopic retrograde cholangiopancreatography, multiple plastic stents, post‐orthotopic liver transplantation

## Abstract

**Objectives:**

Anastomotic biliary strictures are a common complication following orthotopic liver transplantation (post‐OLT), impacting morbidity and graft survival. This meta‐analysis evaluates the efficacy, safety, and cost‐effectiveness of covered self‐expanding metal stents (cSEMS) versus multiple plastic stents (MPS) for treating post‐OLT strictures.

**Methods:**

A systematic review was conducted in PubMed, Cochrane Central, Embase, Scholar, and SciELO. We analyzed randomized controlled trials (RCTs) comparing cSEMS and MPS in post‐OLT biliary strictures. Outcomes included stricture resolution, recurrence, endoscopic retrograde cholangiopancreatography sessions, adverse events, and cost. Standardized mean differences (SMDs) and risk ratios (RRs) were calculated with 95% confidence intervals (CIs). Cost‐effectiveness analysis covered direct and indirect expenses.

**Results:**

Five RCTs with 269 patients were analyzed. No significant differences were found between cSEMS and MPS in terms of stricture resolution (RR = 1.01; 95% CI [0.90, 1.13]; *p =* 0.91), recurrence rates (RR = 2.23; 95% CI [0.74, 6.75]; *p =* 0.15), adverse events (RR = 0.80; 95% CI [0.41, 1.54]; *p =* 0.50), stent migration (RR = 1.55; 95% CI [0.69, 3.50]; *p =* 0.29), number of endoscopic retrograde cholangiopancreatography sessions (SMD = −2.18; 95% CI [−5.28, 0.91]; *p =* 0.12), number of stents (SMD = −2.33; 95% CI [−22.26, 17.59]; *p =* 0.38), treatment time (SMD = −1.60; 95% CI [−4.24, 1.05]; *p =* 0.15), and cost ($10,344 vs. $18,003; *p =* 0.19).

**Conclusion:**

cSEMS and MPS demonstrate similar efficacy and safety for post‐OLT biliary strictures. Both strategies are viable, with selection based on cost, anatomy, and institutional resources.

## 1 Introduction

Biliary strictures are among the most prevalent complications following liver transplantation, leading to significant morbidity and potentially impacting the long‐term success of the transplant [1, 2, 3, 4, 5]. These strictures are broadly categorized into anastomotic and non‐anastomotic, frequently associated with ischemic insults. Managing these complications presents a considerable challenge, with treatment objectives focused on restoring bile flow, minimizing liver damage, and reducing the risk of cholangitis and other severe complications [6, 7, 8].

Endoscopic management, primarily through the placement of stents via endoscopic retrograde cholangiopancreatography (ERCP), has emerged as the preferred approach due to its minimally invasive nature compared to surgical revision [6, 7, 8]. Stents are crucial for maintaining ductal patency, allowing the scar tissue to heal without causing a narrowing of the bile duct. However, the choice between metallic and plastic stents remains a subject of ongoing debate within the medical community [9, 10, 11].

Plastic stents, while cheaper and more readily available, require multiple exchanges and multiple stents. On the other hand, metallic stents, though initially more costly, can remain in place longer and are thought to provide better long‐term patency with fewer interventions [12, 13]. Fully covered self‐expanding metallic stents are designed to prevent the problems of tissue ingrowth that occur with partially covered or uncovered metallic stents [14, 15, 16]. These innovations aim to merge the durability and patency benefits of metallic stents with the removable nature traditionally associated with plastic stents [15, 16, 17, 18]. Additionally, newer designs in metallic stents include features aimed at minimizing migration, a common complication that can lead to severe adverse outcomes [19].

Despite these technological advancements, there is no consensus on the optimal stenting strategy. Clinical trials and previous meta‐analyses have reported conflicting results regarding the efficacy and safety of these two types of stents [14, 20, 21, 22, 23]. This systematic review and meta‐analysis critically evaluate and synthesize the available randomized clinical trial data to compare the overall effectiveness, safety profiles, and cost‐effectiveness of metallic versus plastic stents. The outcomes of interest include stricture resolution rates, recurrence of strictures, complications associated with each stent type, and their respective costs. By providing a comprehensive analysis of these factors, this study aims to offer evidence‐based recommendations for the selection of stent types in the management of post‐orthotopic liver transplantation (post‐OLT) biliary strictures.

## 2 Materials and Methods

We performed this systematic review and meta‐analysis in accordance with the preferred reporting items for systematic reviews and meta‐analyses (PRISMA) guidelines [24]. The study protocol was registered at the International Prospective Register of Systematic Reviews (registration number: CRD42024539541).

### 2.1 Information Sources and Search Strategy

We searched MEDLINE/PubMed, Cochrane Central Register of Controlled Trials (CENTRAL), Embase, Google Scholar, and Scielo until May 2024. Our search was made using the following keywords: post‐liver transplantation, hepatic transplantation, liver grafting, orthostatic liver transplant, stenosis, stents, and ERCP. Detailed search strategies can be found below in Table S1. Additionally, we reviewed the reference lists of all identified randomized controlled trials (RCTs) and previous systematic reviews and meta‐analyses on the topic of relevant RCTs.

### 2.2 Study Selection

In our systematic review and meta‐analysis, we meticulously defined eligibility criteria to evaluate the comparative efficacy of multiple plastic stents (MPS) versus covered self‐expanding metallic stents (cSEMS) in the initial treatment of anastomotic biliary strictures following liver transplantation. We included only RCTs without imposing language restrictions.

### 2.3 Outcomes Assessed

In this meta‐analysis, we evaluated the outcomes of stenting for biliary strictures after liver transplantation, categorizing them into primary and secondary endpoints. The primary outcome was the stricture resolution rate, defined as the absence of symptoms and/or radiological evidence of biliary stricture after stent removal, confirmed by the ability to pass a 10–12 mm extraction balloon through the anastomosis without resistance [14, 22, 23].

Secondary outcomes included stent patency duration, defined as the time period until stent replacement was required due to occlusion. Complications assessed included stent migration, defined as distal (partial or complete) or proximal displacement of the stent confirmed by radiographic or endoscopic assessment. Other adverse events, such as pancreatitis, cholangitis, bleeding, stent occlusion, abdominal pain, fever, perforation, stent embedding, and anesthesia‐related complications or allergic reactions.

We also evaluated the recurrence rate, which was defined as the reappearance of symptoms or radiological evidence of biliary stricture after initial resolution, requiring further intervention. Another secondary outcome was the number of ERCP procedures required to achieve stricture resolution. Additionally, the overall treatment duration was assessed, measured as the time from the initial stenting procedure to the complete resolution of the stricture.

Finally, a cost analysis was performed to compare the economic implications of different stenting strategies. This included direct costs of stent placement, maintenance, management of complications, and reinterventions. For studies reporting costs in different currencies, all values were converted to US dollars using the exchange rate at the time of publication.

### 2.4 Data Extraction

Data extraction was conducted systematically to reduce the risk of bias and ensure the consistency of information collected from each study included in our meta‐analysis. This process was performed independently by two reviewers using a standardized Excel sheet for data extraction, which has been designed and pilot‐tested specifically for this research. Any disagreements between reviewers were resolved through discussion, or if necessary, a third reviewer was consulted. For RCTs which included patients with different causes of biliary strictures, such as post‐OLT or chronic pancreatitis, we exclusively extracted the data for those with post‐OLT biliary strictures when subgroup analysis is provided. If an included RCT reported treatment effects as a median and range or interquartile range, we computed the mean and standard deviation from these values.

### 2.5 Risk of Bias and Evidence Quality

We evaluated the risk of bias in each included study using the revised Cochrane risk‐of‐bias tool [25]. This tool evaluates biases in several domains: randomization process, measurement of the outcome, deviations from intended interventions, missing outcome data, and selection of the reported result. The quality of evidence was analyzed according to the grading of recommendations assessment, development, and evaluation working group (GRADE) approach [25].

To evaluate potential publication bias, funnel plots were visually inspected for asymmetry. This method helps detect whether the effect sizes of smaller studies differ significantly from larger studies, which might suggest a skewness typically associated with publication bias.

### 2.6 Statistical Analysis

We assessed the pooled effect sizes for seven key outcomes related to liver transplantation and associated complications: number of ERCP sessions, number of stents per patient, success rate, recurrence rate, treatment time, stent migration, and adverse events rate. For the quantitative outcomes of the number of ERCP sessions, number of stents per patient, and treatment time, the standardized mean differences (SMDs) were calculated using Hedge's g method, which adjusts for small sample bias in studies [26]. For the binary outcomes, which include success rate, recurrence rate, stent migration, and adverse events rate, we utilized the risk ratios (RRs), pooled using the Mantel‐Haenszel method to provide a robust estimate that considers the study weights and effect sizes.

To quantify the variance among the included studies, we used two different estimators depending on the nature of the outcome variables. The restricted maximum‐likelihood estimator was employed to calculate the between‐study variance τ^2^ for the continuous outcome of ERCP sessions, allowing us to account for sampling variability. For the binary outcomes, the DerSimonian‐Laird estimator was used, which is well‐suited for synthesizing effect sizes across diverse study conditions and designs [27].

The heterogeneity among studies was quantitatively evaluated using the I^2^ statistic, which describes the percentage of total variation across studies that is due to heterogeneity rather than chance. Values of *I* ^2^ were interpreted on a scale from low to very high (*I* ^2^ <30%: low, 30%–60%: moderate, 61%–75%: high, >75%: very high). Depending on the level of detected heterogeneity, appropriate models were chosen for the meta‐analysis. For low to moderate heterogeneity, fixed‐effect models were used, assuming that the studies share a common effect size. Conversely, in the presence of high or very high heterogeneity, random‐effect models were implemented to accommodate the variance between study effects. In addition, we used the leave‐one‐out model to perform sensitivity analysis to investigate whether a particular RCT contributed the most to this high heterogeneity and to explore whether our results would change after omitting this RCT.

The results are presented as pooled effect sizes with corresponding 95% confidence intervals (CIs) for each outcome. This approach provides a clear and statistically justified summary of the effects observed, allowing for direct comparisons across the different outcomes and treatments considered in this meta‐analysis. In cases where an included study involved multiple benign etiologies, not exclusively post‐transplant cases, and isolating data specific to post‐transplant cases was not feasible, a sensitivity analysis was conducted using a leave‐one‐out approach.

The findings are interpreted in the context of their clinical significance and the robustness of the evidence, considering both statistical results and the clinical contexts of the included studies. Statistical analyses were carried out using R version 4.3.1[28].

## 3 Results

### 3.1 Literature Search Results

Our meta‐analysis synthesized data from five RCTs, involving a total of 269 patients with anastomotic post_OLT biliary strictures. Our search yielded records from several databases, including MEDLINE (*n* = 1619), Embase (*n* = 2171), Google Scholar (*n* = 1210), Cochrane Library (*n* = 79), and SciELO (*n* = 9).

**FIGURE 1:**
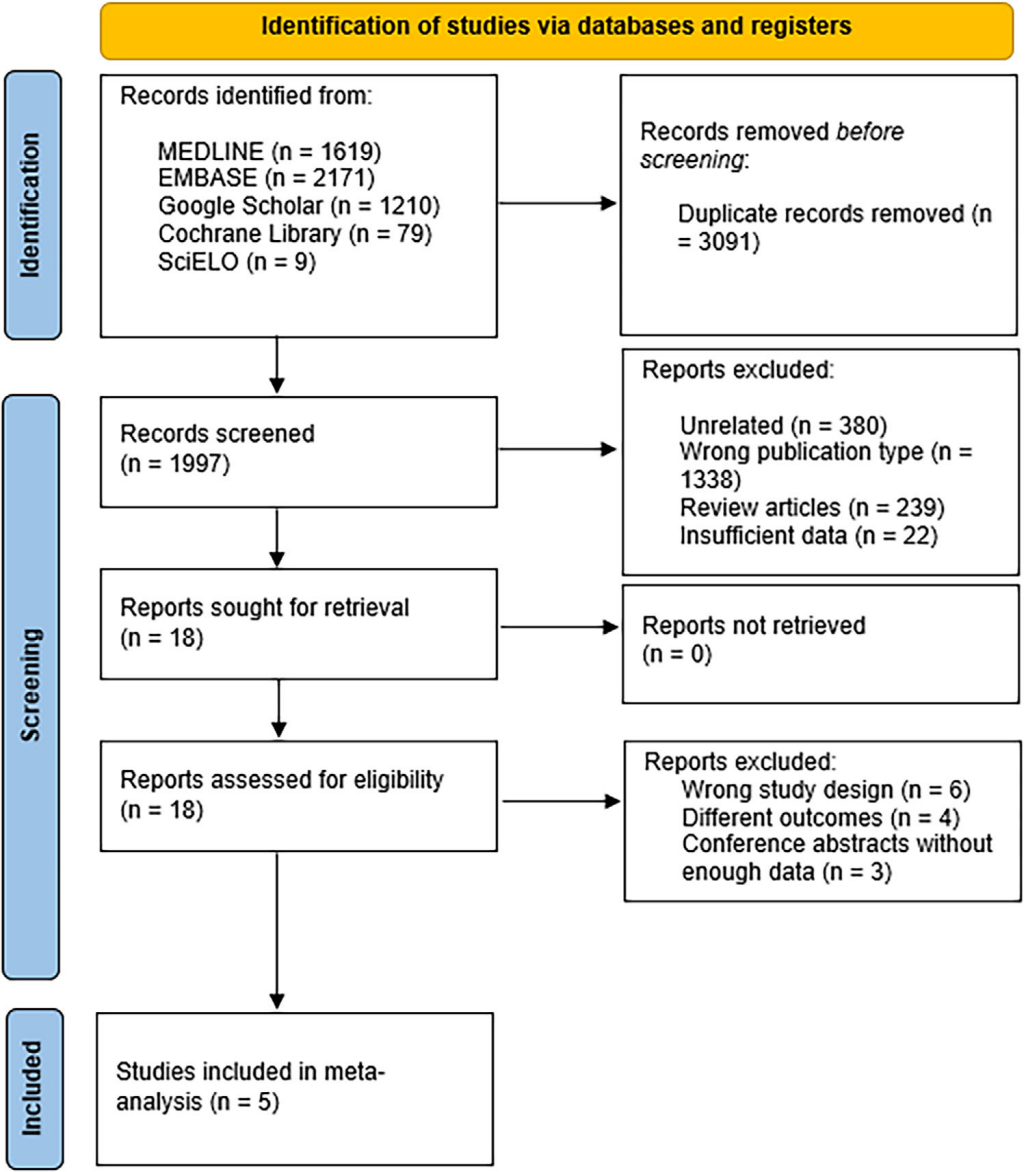
Preferred reporting items for systematic reviews and meta‐analyses (PRISMA) flow diagram for the article selection process, illustrating the systematic review steps from database searching to the final inclusion of studies in the meta‐analysis.

Figure 1 shows the PRISMA flowchart for the article selection process, which was conducted as per the updated guideline.

### 3.2 Characteristics of Included Studies

The demographic data were extracted from the five included RCTs. The median age of patients across these studies ranged from 49.5 to 58.5 years, reflecting a middle‐aged to older adult population predominantly affected by post‐OLT biliary stricture. Gender distribution was relatively balanced in some studies, such as those by Kaffes et al.,[21] where the ratio was an even 5:5 male to female [21]. However, other studies such as those conducted by Martins et al.,[22] and Tal et al.,[23] showed a male predominance.

These studies collectively analyzed 269 patients, with 136 treated with cSEMS and 133 with MPS. cSEMS were typically left in place for at least 12 weeks as observed in the study by Kaffes et al.,[21] while in the study by Martins et al.,[22] patients were randomized to receive a single cSEMS for 6 months or to MPS placement, with the stents exchanged every 3 months over a 1‐year period to ensure stricture resolution.

As shown in Table 1, the baseline characteristics of the included studies are summarized, detailing patient demographics, stent types, and clinical outcomes. This information provides essential context for evaluating the relative effectiveness of different stenting strategies in managing post‐OLT biliary strictures.

**TABLE 1:** Summary of included studies and their patient's baseline characteristics.

Author	Country	Type of Stent	No. of patients	Sex (M/F)	Age, years	Etiology (AS/CP/BI)	ERCP Sessions	Success rate (%)	Treatment duration (months)	No. of stents used per patient	Recurrence	Stent migration	Adverse events	Follow‐up time (months)	COST
Kaffes et al., 2014	Australia	MPS	10	5/5	49.5 (23–69)	10/0/0	4.0 ± 1.17	8/10 (80%)	12	4.0 ± 1.17	3/8	1	5	25.5 (3–44)	$29,280
		cSEMS	10	5/5	56.5 (38–67)	10/0/0	2.0 ± 0.20	10/10 (100%)	3	2.0 ± 0.20	3/10	0	1	26 (6–40)	$12,913
Cote et al., 2016	USA	MPS	55	37/18	56.7 (45–67)	36/17/2	3.13 ± 0.88	41/48 (85.4%)*	12	3.13 ± 0.88	2/41	9	11	24	NR
		cSEMS	57	38/19	54.5 (45–65)	37/18/2	2.21 ± 0.48	50/54 (92.5%)**	6	2.21 ± 0.48	7/50	14	11	24	NR
Tal et al., 2017	Europe	MPS	24	18/6	58.5 (32–72)	24/0/0	5.75 ± 2.61	23/24 (95.8%)	12	5.75 ± 2.61	5/23	0	2	16.9 (2–39.4)	NR
		cSEMS	24	14/10	57 (32–69)	24/0/0	2.0 ± 0.20	24/24 (100%)	6	2.0 ± 0.20	5/24	8	0	13.3 (6.3–34.9)	NR
Martins et al., 2018	Brazil	MPS	29	20/9	50 (28–71)	29/0/0	4.9 ± 0.60	28/29 (96.5%)	12	4.9 ± 0.60	0/28	4	4	32.9	$16,095
		cSEMS	30	22/8	54 (23–73)	30/0/0	2.0 ± 0.20	25/30 (83.3%)	6	2.0 ± 0.20	8/25	3	12	36.4	$6903
Cantu et al., 2021	Italy	MPS	15	14/1	53 (22–68)	15/0/0	4.5 ± 1.15	14/15 (93.3%)	10	4.5 ± 1.15	1/14	2	6	10 (4–24)	$11 269 ± 2946.46***
		cSEMS	15	12/3	59 (50–67)	15/0/0	4.0 ± 1.76	11/15 (73.3%)	9	4.0 ± 1.76	4/11	5	3	9 (4–26)	12 378.20 ± $7916.62***

Abbreviations: AS, anastomotic stricture; BI, bile duct injury; CP, chronic pancreatitis; cSEMS, covered self‐expandable metallic stents; MPS, multiple plastic stents; NR, not reported.

* AS/CP/BI: 31/8/2.

** AS/CP/BI: 33/15/2.

*** Exchange rate November 2020 (€ to $).

**FIGURE 2:**
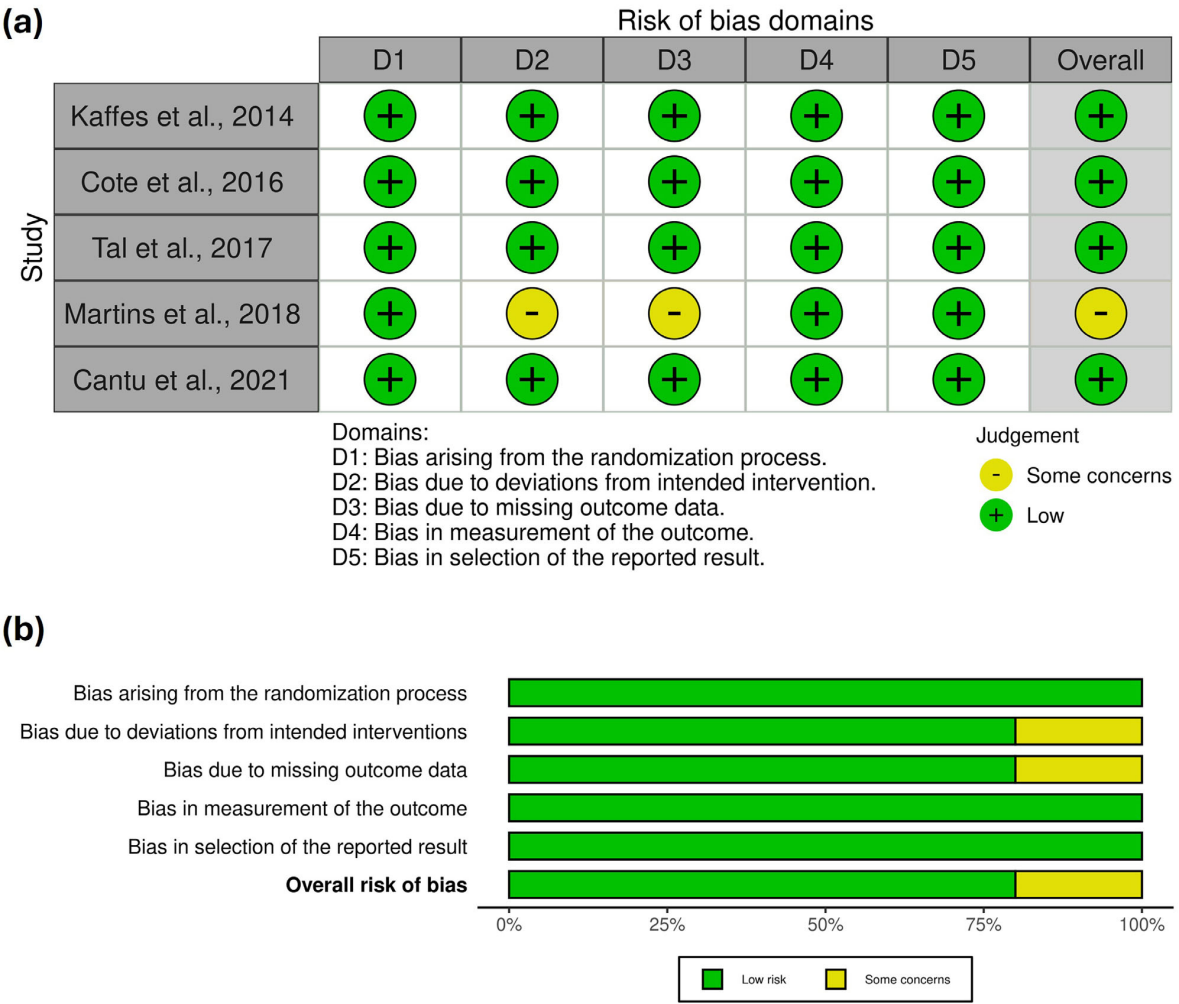
Traffic light plot depicting the risk of bias across the included studies based on the Cochrane risk‐of‐bias (RoB2) tool.

Among the studies, four RCTs had a low risk of bias while one study had some concerns with regards to the risk of bias. The traffic‐light plot for risk of bias is shown in Figure 2.

### 3.3 Outcome Measures

#### 3.3.1 Number of ERCP Sessions

**FIGURE 3:**
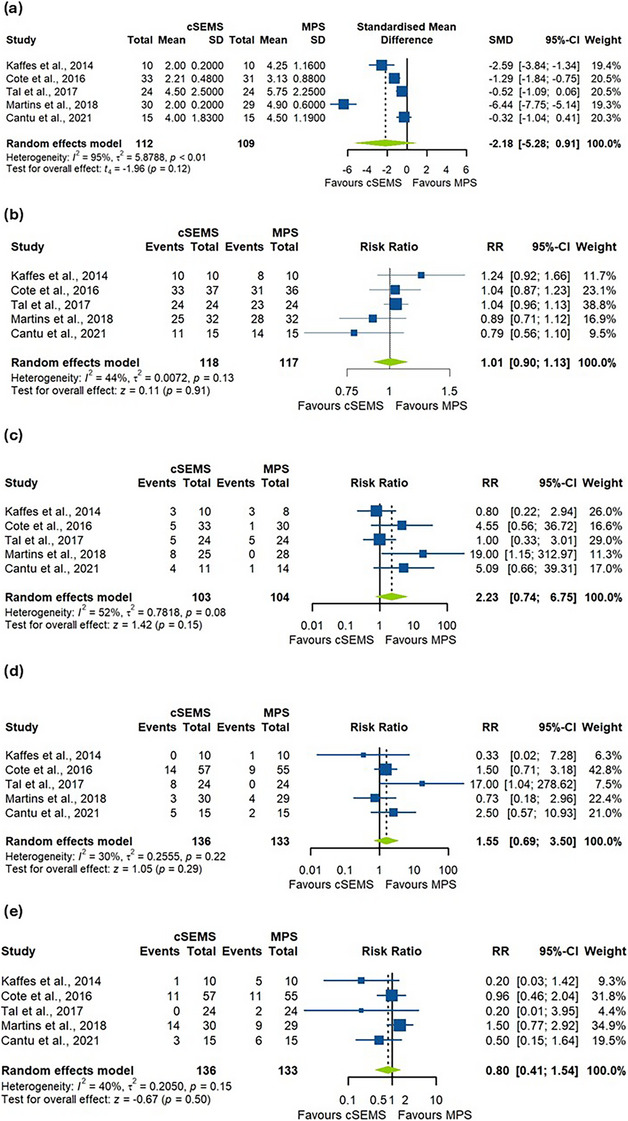
(a) Forest plot for the number of endoscopic retrograde cholangiopancreatography (ERCP) sessions, comparing covered self‐expanding metal stents (cSEMS) and multiple plastic stents (MPS) groups. (b) Forest plot for the stricture resolution rate, showing pooled estimates for both cSEMS and MPS groups. (c) Forest plot for the recurrence rate of strictures between cSEMS and MPS groups. (d) Forest plot for stent migration rates, comparing cSEMS and MPS groups. (e) Forest plot for adverse events, highlighting the comparison between cSEMS and MPS.

Five studies with 221 patients were included in the analysis of the number of ERCPs performed. The number of ERCPs necessary for treatment was comparable between cSEMS and MPS (SMD = −2.18, 95% CI −5.28 to 0.91, *p =* 0.12; Figure 3a). The pooled studies were heterogeneous (*I* ^2^ = 95%, *p* > 0.01). A sensitivity analysis using the leave‐one‐out model showed that this heterogeneity was best decreased by omitting the Martins et al. study and the results remained non‐significant (RR = −1.08, 95% CI −2.59 to 0.44, *I* ^2^ = 77%; Figure S1).

**FIGURE 4:**
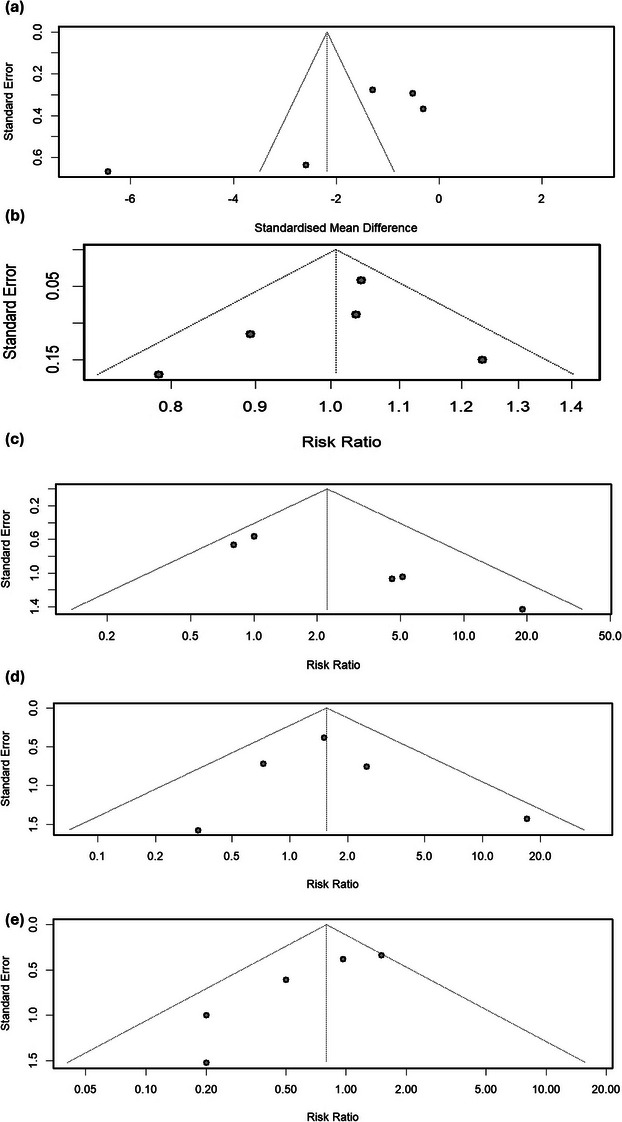
(a) Funnel plot for the number of endoscopic retrograde cholangiopancreatography sessions, assessing potential publication bias among studies. (b) Funnel plot for the stricture resolution rate, evaluating the symmetry of included studies. (c) Funnel plot for the recurrence rate, indicating the distribution of included studies. (d) Funnel plot for stent migration rates, illustrating potential publication bias. (e) Funnel plot for adverse events, showing the symmetry of studies.

The funnel plot in Figure 4a shows that the publications do not follow the shape delineated by the funnel with one study having a very low effect size.

#### 3.3.2 Stricture Resolution

Five studies were included in the analysis of the stricture resolution, with a total of 235 patients. There was no statistically significant difference in the rate of stricture resolution between the two groups (RR = 1.01, 95 % CI 0.90 to 1.13, *p =* 0.91; Figure 3b). The pooled studies were homogenous (*I* ^2 ^= 44%, *p =* 0.13). The funnel plot in Figure 4b shows that the publications follow the shape delineated by the funnel.

#### 3.3.3 Recurrence of Stricture

There was no statistically significant difference found between the two groups in the evaluation of the rate of recurrence in the five studies consisting of 234 patients who had successful initial stricture treatment (RR = 2.23, 95% CI 0.74 to 6.75, *p =* 0.15; Figure 3c). There was moderate heterogeneity among the included RCTs (*I* ^2^ = 52%, *p =* 0.08). A sensitivity analysis using the leave‐one‐out model showed that this heterogeneity was best resolved by omitting Martins et al.’s study, with results remaining non‐significant (RR = 1.51, 95% CI 0.62 to 3.65, *I* ^2^ = 26%; Figure S2). The funnel plot in Figure 4c shows that the publications follow the shape delineated by the funnel.

#### 3.3.4 Stent Migration

There was no statistically significant difference found between the two groups in stent migration rate in the five studies consisting of 269 patients who had the interventions (RR = 1.55, 95 % CI 0.69 to 3.50, *p =* 0.29; Figure 3d). The pooled RCTs were homogenous (*I* ^2^ = 30%, *p =* 0.22). The funnel plot in Figure 4d shows that the publications follow the shape delineated by the funnel.

#### 3.3.5 Number of Stents Per Patient

Two studies, comprising 112 patients, were included in the analysis of the number of stents required per patient. The results indicated that the number of stents per patient was comparable between cSEMS and MPS groups (SMD = −2.33; 95% CI −22.26, 17.59, *p =* 0.38; Figure S3). A high heterogeneity was observed in this analysis (*I* ^2^ = 97%, *p *> 0.01).

#### 3.3.6 Treatment Time

Four studies consisting of 205 patients were included in the analysis of treatment time. The Treatment time was comparable between the cSEMS and MPS groups (SMD = −1.60, 95% CI −4.24 to 1.05, *p =* 0.15; Figure S4). The pooled studies were heterogeneous (*I* ^2^ = 95%). A sensitivity analysis using the leave‐one‐out model showed that this heterogeneity was slightly decreased by omitting the Martins et al. study and the results remained non‐significant (RR = −0.97, 95% CI −4.45 to 2.52, *I* ^2^ = 86%; Figure S5). The funnel plot in Figure S6 shows that the publications do not follow the shape delineated by the funnel except for one study.

#### 3.3.7 Adverse Events

Five studies were included in the analysis of adverse events not related to stent migration, with a total of 269 patients. No statistically significant difference was observed (RR = 0.80, 95% CI 0.41 to 1.54, *p =* 0.50; Figure 3e). The pooled studies were homogeneous (*I* ^2^ = 40%, *p =* 0.15). The funnel plot in Figure 4e shows that the publications follow the shape delineated by the funnel.

#### 3.3.8 Cost‐Effectiveness Analysis

**FIGURE 5:**
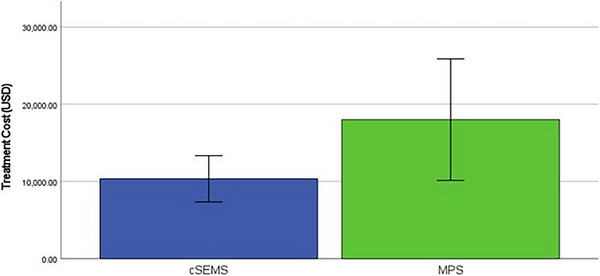
Cost‐effectiveness analysis between covered self‐expanding metal stents (cSEMS) and multiple plastic stents (MPS) treatments, converted to US dollars for comparison.

Three studies reported the average cost of each treatment. Kaffes et al. reported the cost in Australian dollars. Cantu et al. reported it in Euro, while Martins et al. reported it in US dollars. We converted all costs to US dollars using the exchange rate at the time of each study. The cost analysis showed no statistically significant difference in cost between the treatment with cSEMS and MPS ($10 344 ± 2996 vs. $18 003 ± 7863; *p* = 0.19; Figure 5).

### 3.4 Grade of Evidence

Table 2 presents our findings based on the GRADE approach. According to the GRADE approach, the strength of our evidence was high for success rate, moderate for stent migration, low for the number of ERCP sessions, recurrence rate, and adverse events, and very low for treatment time and number of stents per patient.

**TABLE 2:** The certainty of evidence using the Grading of Recommendations Assessment, Development, and Evaluation (GRADE) framework.

Outcome	No. of participants (Studies)	Risk of bias	Inconsistency	Indirectness	Imprecision	Publication bias	Others	Effect estimate (95% CI)	Overall certainty of evidence
ERCP sessions	221 (five RCTs)	No downgrade	Downgrade by one	No downgrade	Downgrade by one	No downgrade	None	SMD = −2.18 (−5.28 to 0.91)	Low ⊕⊕⊖⊖
Success rate	235 (five RCTs)	No downgrade	No downgrade	No downgrade	No downgrade	No downgrade	None	RR = 1.01 (0.90 to 1.13)	High ⊕⊕⊕⊕
Recurrence rate	207 (five RCTs)	No downgrade	No downgrade	No downgrade	Downgrade by one	Downgrade by one	None	RR = 2.23 (0.74 to 6.75)	Low ⊕⊕⊖⊖
Stent migration	269 (five RCTs)	No downgrade	No downgrade	No downgrade	Downgrade by one	No downgrade	None	RR = 1.55 (0.69 to 3.50)	Moderate ⊕⊕⊕⊖
Adverse events	269 (five RCTs)	No downgrade	No downgrade	No downgrade	Downgrade by one	Downgrade by one	None	RR = 0.80 (0.41 to 1.54)	Low ⊕⊕⊖⊖
Treatment time	205 (four RCTs)	No downgrade	Downgrade by one	No downgrade	Downgrade by one	Downgrade by one	None	RR = −1.60 (−4.24 to 1.05)	Very low ⊕⊖⊖⊖
Stents per patients	112 (two RCTs)	No downgrade	Downgrade by one	No downgrade	Downgrade by one	Downgrade by one	None	RR = −2.33 (−22.26 to 17.59)	Very low ⊕⊖⊖⊖

*Note*: ⊕⊕⊕⊕ High certainty; ⊕⊕⊕⊖ Moderate certainty; ⊕⊕⊖⊖ Low certainty; ⊕⊖⊖⊖ Very low certainty — based on the GRADE approach.

Abbreviations: CI, confidence interval; GRADE, Grading of Recommendations Assessment, Development, and Evaluation; RR, risk ratio; SMD, standardized mean difference.

## 4 Discussion

In this meta‐analysis, we systematically investigated the efficacy and safety of two primary stenting strategies, cSEMS and MPS in the management of anastomotic post‐OLT biliary strictures. Our findings indicate that both stenting methods are comparably effective in achieving stricture resolution. This lack of significant difference suggests that the choice between cSEMS and MPS may be based on other factors such as cost, availability, and physician preference, rather than efficacy alone. Even though the cSEMS treatment involves only two procedures, one to insert the stent and another to remove it, we found that the number of ERCP sessions was comparable between cSEMS and MPS groups.

The recurrence rates of biliary strictures, a key outcome for patient prognosis, showed no statistically significant difference between cSEMS and MPS in our primary meta‐analysis, aligning with previous studies. Martins et al. reported a higher recurrence rate in the cSEMS group, hypothesizing that the shorter dwell time of cSEMS (6 months) compared to MPS (12 months) contributed to this difference. Notably, six of the eight cases of recurrence in the cSEMS group were successfully resolved with subsequent MPS treatment for one year. Similarly, Cantù et al. found no significant differences in recurrence rates but observed that some patients initially treated with cSEMS required conversion to MPS to achieve resolution, highlighting the potential need for individualized management strategies.

However, when considering variations in follow‐up durations across studies, our time‐to‐event analysis revealed a significantly lower recurrence risk in the cSEMS group compared to the MPS group (pooled HR = 0.26, 95% CI 0.11–0.65). This suggests that, over time, patients treated with cSEMS may have a more durable stricture resolution, possibly due to prolonged luminal patency and reduced need for multiple interventions. These findings highlight the importance of considering time‐dependent recurrence risk rather than absolute event rates alone. Further long‐term studies with standardized follow‐up protocols are needed to confirm these observations.

Follow‐up duration varied significantly across studies, potentially influencing recurrence data. While studies like those by Cote et al., Martins et al., and Tal et al. included a minimum follow‐up of 12 months, Kaffes et al. did not report follow‐up durations, and Cantù et al. provided longer‐term follow‐up of 60 months. This variability underscores the need for standardized protocols and extended follow‐up periods to better assess long‐term recurrence, particularly in the cSEMS group where data remain limited.

The adverse events, a critical consideration in choosing a stenting strategy, were comparable for both cSEMS and MPS. The similarity in the incidence of complications suggests that both stents are safe for clinical use. Similarly, stent migration, a specific adverse event that can significantly impact patient outcomes, showed no statistically significant difference between the two stent types. This finding underscores the improvements in stent design that have minimized the risks of migration, thus enhancing the reliability of both cSEMS and MPS in clinical practice [19].

Our findings align with those of Visconti et al. in terms of success rates, stricture recurrence, and adverse events (Table S2). However, discrepancies arise regarding the number of ERCP sessions, number of stents per patient, treatment duration, and cost analysis. Importantly, our meta‐analysis incorporates the recent study by Cantù et al., which was not included in the analysis by Visconti et al., thereby providing a more comprehensive body of evidence. Cantù et al. reported better outcomes with MPS; however, a notable proportion of participants in the cSEMS group required continued treatment with plastic stents due to clinical failure. This higher reintervention rate in the cSEMS group may have been influenced by stricter criteria for reintervention and a higher incidence of stent migration (29% vs. 2.6% in MPS).

Although the study by Cantù et al. provides valuable insights into the economic advantages of cSEMS in patients with clinical success, reporting a 41% cost reduction compared to MPS in this subgroup, this finding could not be corroborated in our meta‐analysis, as our results showed no statistically significant difference in overall costs between the two stenting strategies. Furthermore, the lack of comparable data from other included studies limits the ability to perform a quantitative subgroup analysis. Most studies report overall costs without stratifying by clinical success, making it difficult to assess whether similar economic benefits are observed across different settings. Future studies should aim to provide detailed economic analyses stratified by clinical outcomes to facilitate more robust evaluations of cost‐effectiveness in this population.

Despite the robust methodology employed in this meta‐analysis, certain limitations must be acknowledged, as they may influence the interpretation of the findings. First, the relatively small number of included RCTs reduces the statistical power to detect subtle differences between cSEMS and MPS, particularly for secondary outcomes such as the number of ERCP sessions and adverse events. This limitation is further compounded by the high heterogeneity observed in certain outcomes, including treatment duration and the number of stents per patient, which reflects variability in procedural techniques, stent dwell times, and definitions of clinical success across the included studies.

Moreover, although a time‐dependent analysis would provide a more accurate estimate of recurrence risk across variable follow‐up durations, none of the included studies reported Kaplan–Meier curves or individual hazard ratios. Consequently, a hazard ratio meta‐analysis could not be performed, and the risk ratio remains the most appropriate method for comparing recurrence outcomes in this context. Furthermore, the incidence of bile duct strictures following living donor liver transplantation has decreased in recent years due to advancements in surgical techniques, perioperative management, and postoperative surveillance. This reduction in case frequency has limited the number of eligible participants for randomized controlled trials, thereby posing an additional challenge for conducting robust comparative analyses.

Another important source of variability among the included studies is the schedule for programmed stent exchanges. While most studies utilizing MPS adopted a 3‐month interval for stent replacement, Tal et al. implemented shorter intervals of 6–12 weeks. Similarly, the dwell times for cSEMS ranged from 12 weeks in the study by Kaffes et al. to 6 months in Martins et al., Cantu et al., and Coté et al. These inconsistencies may have influenced key outcomes such as the number of ERCP sessions, treatment duration, and complication rates, particularly stent migration. Together, these limitations highlight the need for future studies with larger sample sizes, standardized protocols, and consistent definitions of outcomes to reduce heterogeneity and improve the applicability of the results in diverse clinical settings.

In conclusion, this meta‐analysis confirms the comparable efficacy and safety of cSEMS and MPS in managing post‐OLT biliary strictures, supporting their interchangeable use based on patient‐specific factors and healthcare resources. While heterogeneity remains a challenge, our findings provide updated evidence to inform clinical decision‐making and highlight the need for standardized protocols and cost‐effectiveness analyses in future studies.

## 5 Conclusion

Our findings demonstrate that cSEMS and MPS offer comparable outcomes in the management of post‐OLT biliary strictures under a protocol of scheduled stent exchange. Both stent types showed similar efficacy in stricture resolution, as well as equivalence in the number of ERCP sessions, stents required per patient, stent migration rates, and adverse event profiles. These results highlight the reliability of both strategies, enabling healthcare providers to select the most appropriate option based on additional factors such as cost, patient‐specific anatomical considerations, and institutional resources.

## Ethics Statement

This study is a systematic review and meta‐analysis of previously published randomized controlled trials. As such, it did not involve direct participation of human subjects or animals and did not require ethical approval or informed consent.

## Conflict of Interest

The authors declare no conflicts of interest.

## Patient Consent Statement

The authors have nothing to report.

## Clinical Trial Registration

The authors have nothing to report.

## Supporting information




**Supplementary Table 1**: Full search strategy for each database.
