# Supplementary material for: Covered Self‐Expanding Metal Stents Versus Multiple Plastic Stents in Treating Biliary Strictures Post‐Orthotopic Liver Transplantation: A Systematic Review and Meta‐Analysis of Randomized Controlled Trials
Source: DEN Open. 2025 May 23;6(1):e70143. doi: 10.1002/deo2.70143 (PMC12101909; doi:10.1002/deo2.70143)
Supplement: Supplementary file 1 — Supplementary Table 1: Full search strategy for each database. [file DEO2-6-e70143-s001.docx]

**Supplementary materials**

**Supplementary Table 1:** Full search strategy for each database.

| Database | Search Query | Search field | Search results |
| --- | --- | --- | --- |
| Embase | ('liver transplantation'/exp OR 'liver transplant':ab,ti OR 'hepatic transplantation':ab,ti) AND ('anastomosis complication'/exp OR 'anastomotic stricture'/exp OR 'stricture':ab,ti OR 'stenosis':ab,ti) AND ('stent'/exp OR 'endoscopic retrograde cholangiopancreatography'/exp OR 'ERCP':ab,ti OR 'endoscopy'/exp) |  | 2171 |
| Google Scholar | "Benign bile duct stricture" OR "anastomotic bile duct stricture" OR "Biliary stricture" OR "Chronic pancreatitis related bile duct stricture" AND "self-expandable metallic stent" OR "SEMS" OR "metal stent" OR "plastic stents" OR "Multiple plastic stent" AND "liver stricture" OR "liver transplant" |  | 1210 |
| Scielo | ("Liver transplantation" OR "Hepatic transplantation") AND ("Anastomosis" OR "Pathological constriction" OR "Stricture" OR "Stenosis") AND ("Stents" OR "Endoscopic Retrograde Cholangiopancreatography" OR "ERCP" OR "Endoscopy") |  | 9 |
| MEDLINE | (Post-liver transplantation OR Liver transplantation OR Liver transplant OR Hepatic Transplantation OR Liver Grafting OR Orthostatic liver transplant) AND (Anastomosis, Surgical/adverse effects OR Constrictions, Pathologic OR Stricture* OR Stenose* OR Stenosis*) AND (Prosthesis Implantation/instrumentation* OR Stents* OR Cholangiopancreatography, Endoscopic Retrograde OR ERCP OR Cholangiography OR Endoscopic Retrograde Cholangiopancreatographies OR Endoscopy, Surgical OR Endoscopic Surgical Procedure OR Endoscopic Surgical Procedures OR Endoscopy) |  | 1619 |
| Cochrane Library |  |  | 79 |

**Supporting information legend:** Full search strategies for each database, including the database name, search query, search fields, and the number of search results obtained. The databases included are Embase, Google Scholar, Scielo, MEDLINE, and Cochrane Library.

**Supplementary Table 2** Comparison of our study with the previous three meta-analyses

|  | Khan et al. 2017 | Visconti et al. 2018 | Giri et al. 2022 | Our study |
| --- | --- | --- | --- | --- |
| Etiology of biliary stricture | Chronic pancreatitis and post-OLT | Post-OLT | Chronic pancreatitis and post-OLT | Post-OLT |
| Number of RCTs with post-OLT patients | 2 RCTs | 4 RCTs | 5 RCTs | 5 RCTs |
| Subgroup analysis based on the etiology of biliary stricture | No | NA | For stricture resolution only | NA |
| Used Hedge's g adjustement | No | No | No | Yes |
| Performed sensitivity analysis | No | No | No | Yes |
| Analyzed cost-effectiveness | No | Yes | No | Yes |
| Number of ERCP sessions | Lower with cSEMS | Lower with cSEMS | Lower with cSEMS | ND |
| Stricture resolution | ND | ND | ND | ND |
| Recurrence of stricture | ND | ND | ND | ND |
| Stent migration | NR | ND | ND | ND |
| Treatment time | NR | Lower with cSEMS | NR | ND |
| Number of stents per patient | NR | Lower with cSEMS | NR | ND |
| Adverse events | ND | ND | ND | ND |

Abbreviations: OLT, orthotopic liver transplant; RCTs, randomized controlled trials; ERCP, endoscopic retrograde cholangiopancreatography; NA, not applicable; ND, no difference; NR, not reported; cSEMS, self-expandable metallic stent

**Supporting information legend:**: Comparison of the current study with previous meta-analyses, highlighting key aspects such as etiology of biliary structure, number of RCTs included, use of fixed/flexible models, subgroup analyses, statistical adjustments, and various outcomes like adverse events, stent migration, and recurrence rates.

**Supplementary Figure 1** Sensitivity analysis for the number of ERCP sessions using the leave-one-out model,

**
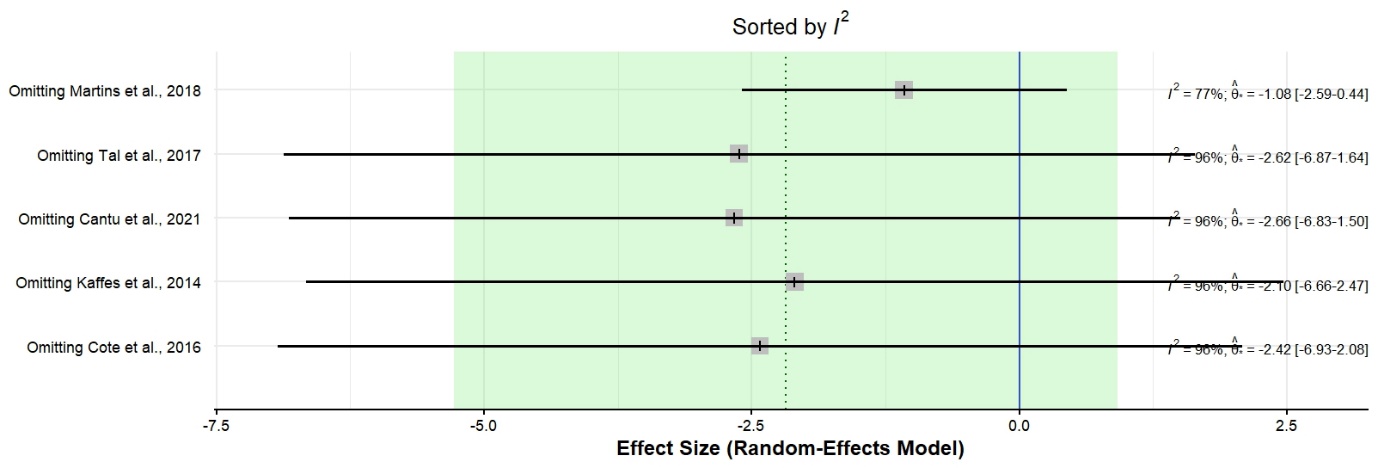
**

**Supporting information legend:** Sensitivity analysis for the number of ERCP sessions using the leave-one-out model, visualizing the effect of excluding individual studies on the overall results.

**Supplementary Figure 2** Sensitivity analysis for the recurrence of stricture using the leave-one-out model.

**
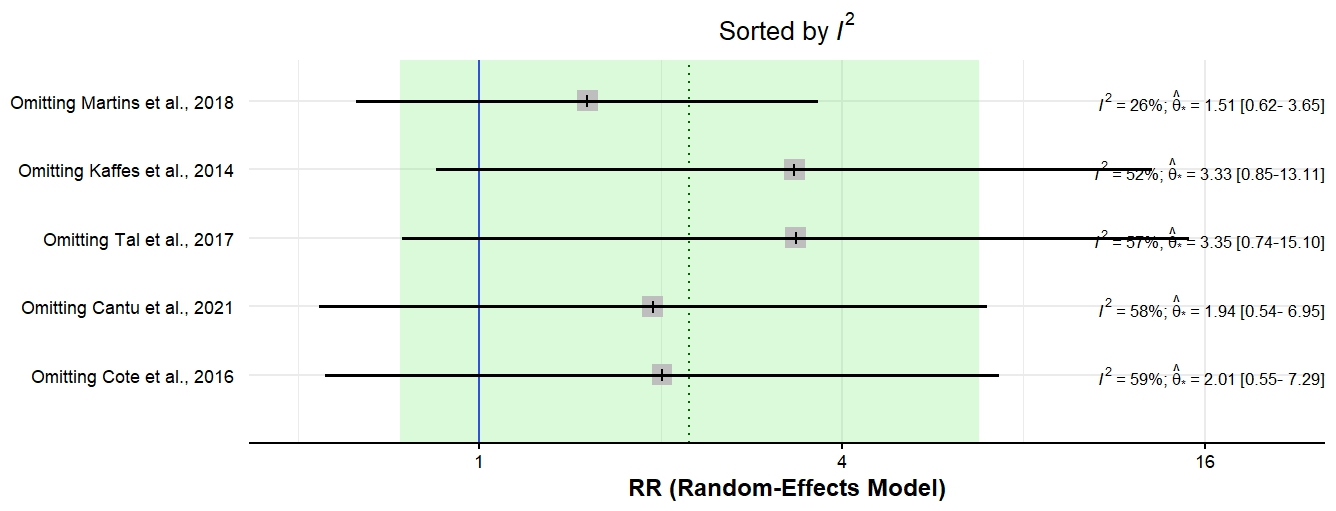
 Supporting information legend:** Sensitivity analysis for the recurrence of stricture using the leave-one-out model, demonstrating the effect of excluding individual studies on the pooled recurrence rate.

**Supplementary Figure 3** Forest plot for the number of stents per patients.

**
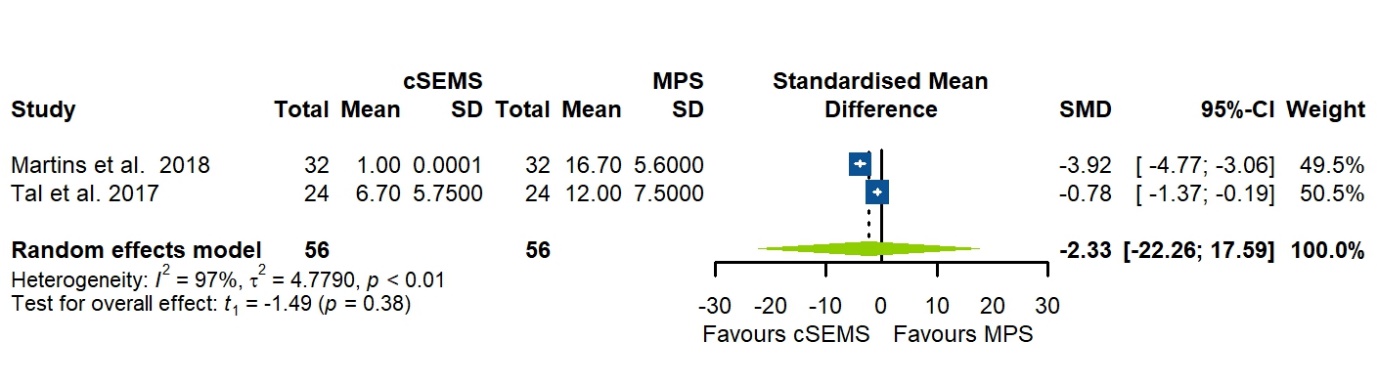
Supporting information legend:** Forest plot showing the number of stents per patient, comparing outcomes between groups and highlighting the standardized mean differences.

**Supplementary Figure 4** Treatment time forest plot.

**
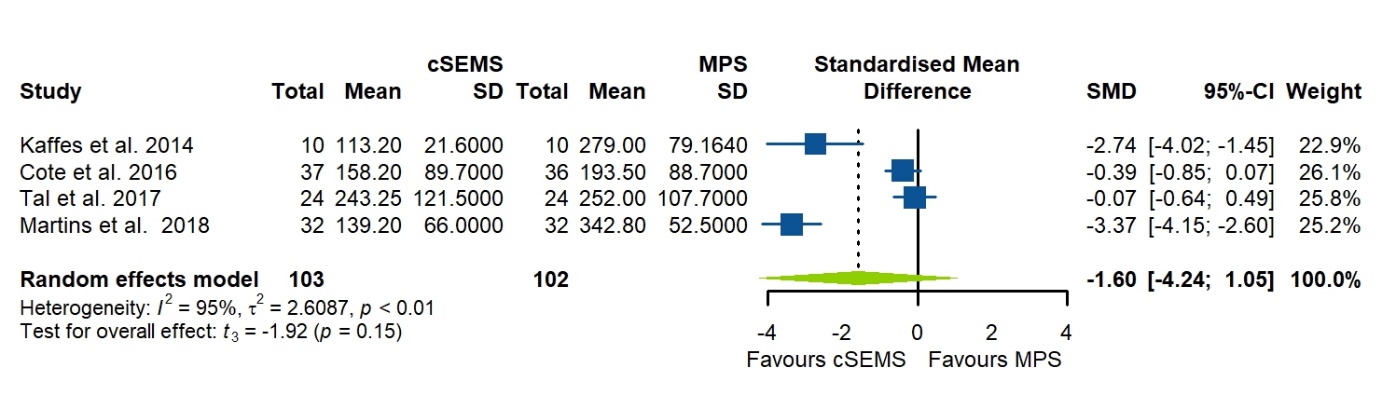
 Supporting information legend:** Treatment time forest plot, illustrating the comparison between groups and their respective standardized mean differences.

**Supplementary Figure 5** Sensitivity analysis for the treatment time using the leave-one-out model.


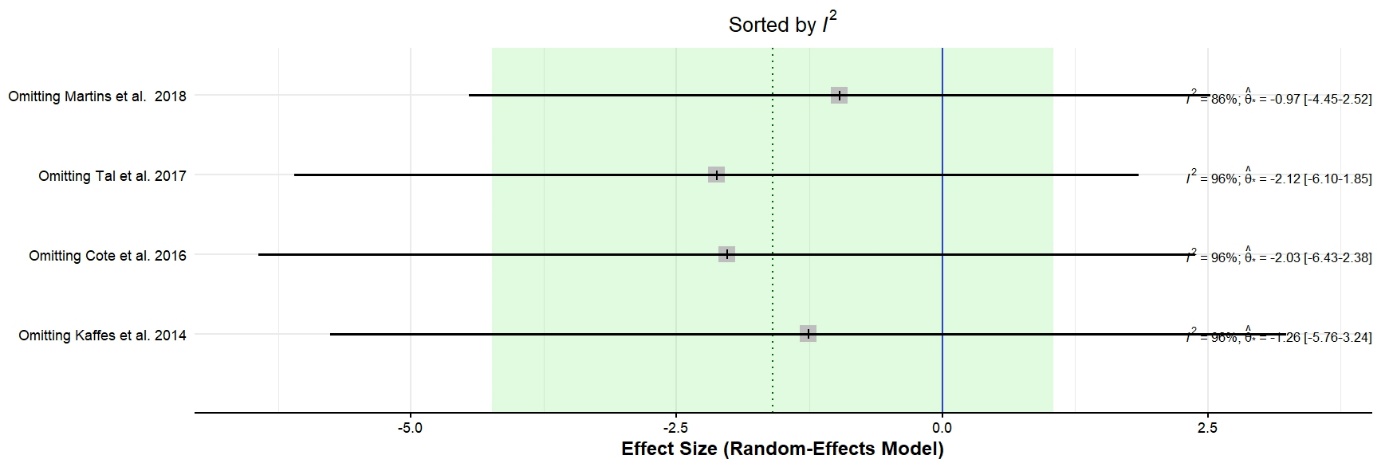


**Supporting information legend:** Sensitivity analysis for treatment time using the leave-one-out model, assessing the influence of individual studies on the overall treatment time estimate.

**Supplementary Figure 6** Funnel plot for treatment time.**
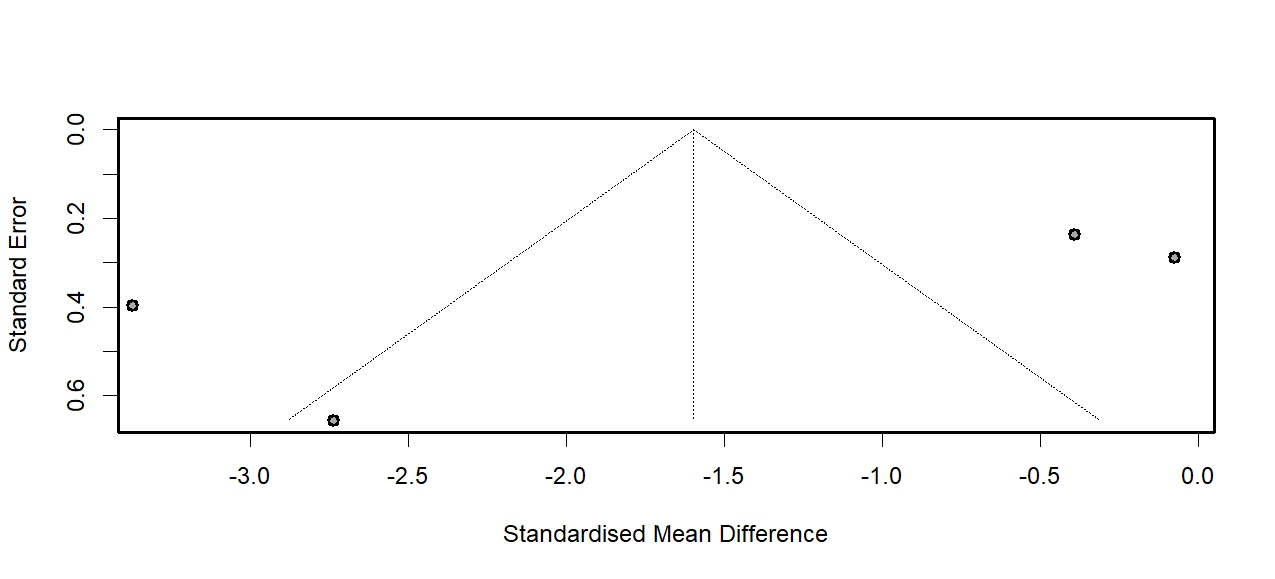
**

**Supporting information legend:** Funnel plot for treatment time, evaluating potential publication bias by visualizing the distribution of effect sizes and their standard errors across studies.
